# Breastfeeding and treatment of multiple sclerosis

**DOI:** 10.1177/1352458520931786

**Published:** 2020-06-08

**Authors:** Elisabeth G Celius

**Affiliations:** Department of Neurology, Oslo University Hospital, Oslo, Norway; Institute of Clinical Medicine, University of Oslo, Oslo, Norway

Multiple sclerosis (MS) is a disease with onset around 30 years of age, coinciding with the age when females are giving birth to their first child. Over the last few years, healthy females have fewer children; females with MS are having more children.^1^ In patients with highly active disease, breastfeeding may not be sufficient to prevent relapses postpartum,^2^ and the reinitiating of potent disease modifying drugs is usually indicated. The majority of the available disease modifying drugs is contraindicated either due to known potential effects in the baby or lack of knowledge about transfer to breast milk or lack of knowledge about possible uptake in the baby.^3^ Even in drugs that have been available for years like natalizumab and rituximab, there is lack of knowledge.^4^ As it is unethical to perform clinical trials in pregnant and breastfeeding patients, collection of evidence through case reports and case series is how clinical practice and prescribing information may be updated. The present study by Datta et al. is an important contribution to our knowledge about the extent and duration of transfer of cladribine into breast milk.^5^ With studies confirming their findings, the period of stopping breastfeeding after a treatment course of cladribine may possibly be reduced from 7 to 1 or 2 days after each treatment cycle. Further studies on cladribine and also the other MS-treatments are urgently needed to help us guide the increasing number of female MS-patients wishing to breastfeed their babies.
